# Diseases of Women

**Published:** 1904-09

**Authors:** 


**Diseases of Women.** By GEORGE ERNEST HERMAN, M.B. Revised Edition. Pp. xvi., 884. London: Cassell and Company, Limited. 1903.

The author describes his work as a clinical guide to the diagnosis and treatment of diseases of women, and states in the preface that he has endeavoured to present these affections in the way in which they appear in practice. As a clinical guide the work is most reliable, and the manner in which the author presents his subject is thoroughly practical.

We do not feel sure, however, that for the student, looked at as an examinee whose opportunities of gaining practical experience have been rather limited, the book is entirely suitable, since it deals very largely with conditions which are not obtruded strongly on the notice of those not in practice.

To the practitioner, however, we consider the information contained in the book will be most valuable; the author deals in clear and succinct language with the various affections indicated by such symptoms as "headache," "abdominal pain," "neurasthenia," &c., and gives most valuable hints, gathered from his own extensive clinical experience, as to the treatment of the various affections underlying these symptoms. His remarks as to the various forms of sexual disturbance are particularly practical, and will perhaps do something to dissipate certain opinions held in the profession which we entirely agree with the author are not only exaggerated but erroneous.

The work throughout is stamped by the author's originality and strong common sense; he does not adduce any startling novelties as to treatment, but by sound pathological reasoning shows the extent to which different methods can be relied on. His remarks on "stem pessaries," "dysmenorrhœa," "endometritis," are particularly worthy of perusal, to instance simple affections; while his views on the early symptoms of malignant disease, the treatment of fibroids, &c., as instances of the more complicated conditions, should meet with universal approval and attention.

The work is not in the least dry reading; in fact, we consider it one of the most readable manuals we have met with, and have no hesitation in saying that no practitioner of whatever standing will read it without profit, while those whose clinical experience is not yet extensive will find it most valuable.

